# Supplementary material for: Past Happiness and Broken Future Horizon of Oncological Patients during Chemotherapy—A Quantitative Exploration of a Phenomenological Hypothesis
Source: Cancers (Basel). 2024 Jun 2;16(11):2124. doi: 10.3390/cancers16112124 (PMC11171201; doi:10.3390/cancers16112124)
Supplement: Supplementary file 1 [file cancers-16-02124-s001.zip › cancers-3002634-supplementary.pdf]

Metryczka

Wiek ......

Płeć:

a) mężczyzna b) kobieta c) inne………

Wykształcenie:

a) podstawowe; b) zawodowe; c) średnie; d) wyższe licencjackie; e) wyższe magisterskie; f) wyższe doktorskie; g) wyższe medyczne

Miejsce zamieszkania:

a) miejscowość do 5.000; b) miejscowość do 20.000; c) miejscowość do 100.000; d) miejscowość do 250.000; e) miejscowość do 500.000; f) miejscowość powyżej 500.000

Dochód na rękę:

a) do 1000 PLN; b) do 2000 PLN; c) do 3000 PLN; d) do 5000 PLN; e) powyżej 5000 PLN

Rodzaj nowotworu (np. jajnika, płuc, jelita grubego, piersi): ……………………………………….

Czas, który minął od postawienia diagnozy: …………………………………………........................

Czas, który minął od rozpoczęcia chemioterapii: …………………………………….

Aktualna częstotliwość kursów chemioterapii:

a) Co tydzień b) Co dwa tygodnie c) Co trzy tygodnie

d) Inne/jakie………
…………………………………………………………

1. Nie myślę o przeszłości bo skupiam się na teraźniejszości

a) Całkowicie się zgadzam
b) Zgadzam się
c) Raczej się zgadzam
d) Nie mam zdania
e) Raczej się nie zgadzam
f) Nie zgadzam się
g) Zdecydowanie się nie zgadzam

2. Myśli o moim życiu przed leczeniem powracają do mnie często w formie żalu – żałuję, że zrobiłam/łem coś, co mogło mieć negatywne konsekwencje:

a) Całkowicie się zgadzam
b) Zgadzam się
c) Raczej się zgadzam
d) Nie mam zdania
e) Raczej się nie zgadzam
f) Nie zgadzam się
g) Zdecydowanie się nie zgadzam

3. Niezależnie od powyższego, myśli o moim życiu przed leczeniem powracają do mnie często w formie szczęśliwych wspomnień:

a) Całkowicie się zgadzam
b) Zgadzam się
c) Raczej się zgadzam
d) Nie mam zdania
e) Raczej się nie zgadzam
f) Nie zgadzam się
g) Zdecydowanie się nie zgadzam

4. Żyję dniem codziennym:

a) Całkowicie się zgadzam
b) Zgadzam się
c) Raczej się zgadzam
d) Nie mam zdania
e) Raczej się nie zgadzam
f) Nie zgadzam się
g) Zdecydowanie się nie zgadzam

5. Gdy wybiegam myślami w przyszłość to nie dalej niż:

a) kolejny dzień
b) kolejny tydzień
c) kolejne podanie chemii
d) kolejne 3 miesiące
e) kolejne pół roku
f) kolejny rok i dłużej

6. Planuję przyszłość nie dalej niż do następnej chemii

a) Całkowicie się zgadzam
b) Zgadzam się
c) Raczej się zgadzam
d) Nie mam zdania
e) Raczej się nie zgadzam
f) Nie zgadzam się
g) Zdecydowanie się nie zgadzam

7. Nie mam konkretnych planów na przyszłość po wyzdrowieniu, co najwyżej marzenia:

a) Całkowicie się zgadzam
b) Zgadzam się
c) Raczej się zgadzam
d) Nie mam zdania
e) Raczej się nie zgadzam
f) Nie zgadzam się
g) Zdecydowanie się nie zgadzam

8. Żyję w ciągłym poczuciu, że może się wydarzyć coś nieprzewidzianego:

a) Całkowicie się zgadzam
b) Zgadzam się
c) Raczej się zgadzam
d) Nie mam zdania
e) Raczej się nie zgadzam
f) Nie zgadzam się
g) Zdecydowanie się nie zgadzam

9. W trakcie leczenia czas mierzę:

a) Rytmem miesięcznym
b) Rytmem tygodniowym
c) Rytmem dziennym
d) Rytmem kursów chemioterapii
e) Rytmem badań kontrolnych
